# The Influence of a Competitive Field Hockey Match on Cognitive Function

**DOI:** 10.3389/fnhum.2022.829924

**Published:** 2022-03-04

**Authors:** Rachel Malcolm, Simon Cooper, Jonathan P. Folland, Christopher J. Tyler, Caroline Sunderland

**Affiliations:** ^1^Department of Sport Sciences, Sport, Health and Performance Enhancement (SHAPE) Research Centre, Nottingham Trent University, Nottingham, United Kingdom; ^2^School of Sport, Exercise and Health Sciences, Loughborough University, Loughborough, United Kingdom; ^3^Department of Life Sciences, University of Roehampton, Roehampton, United Kingdom

**Keywords:** perception, executive function, BDNF, neurobiological changes, catecholamines

## Abstract

Despite the known positive effects of acute exercise on cognition, the effects of a competitive team sport match are unknown. In a randomized crossover design, 20 female and 17 male field hockey players (19.7 ± 1.2 years) completed a battery of cognitive tests (Visual Search, Stroop, Corsi Blocks, and Rapid Visual Information Processing) prior to, at half-time, and immediately following a competitive match (or control trial of seated rest); with effect sizes (ES) presented as raw ES from mixed effect models. Blood samples were collected prior to and following the match and control trial, and analyzed for adrenaline, noradrenaline, brain derived neurotrophic factor (BDNF), cathepsin B, and cortisol. The match improved response times for a simple perception task at full-time (ES = –14 ms; *P* < 0.01) and response times on the complex executive function task improved at half-time (ES = –44 ms; *P* < 0.01). Working memory declined at full-time on the match (ES = –0.6 blocks; *P* < 0.01). The change in working memory was negatively correlated with increases in cortisol (*r* = –0.314, *P* = 0.01; medium), as was the change in simple perception response time and the change in noradrenaline concentration (*r* = –0.284, *P* = 0.01; small to medium). This study is the first to highlight the effects a competitive hockey match can have on cognition. These findings have implications for performance optimization, as understanding the influence on specific cognitive domains across a match allows for the investigation into strategies to improve these aspects.

## Introduction

In open skill sports such as field hockey, skill is determined by a player’s ability to adapt and perform a specific action despite the environmental constraints imposed upon them by calling upon neural resources from changeable regions of the brain (Allard and Burnett, 1987; Starkes, 1987; Williams and Jackson, 2019; Morris-Binelli et al., 2020). With the recently adapted rules, field hockey has become one of the most fast paced team sports, hence the ability to anticipate, adapt and respond successfully relies on superior perceptual-cognitive factors (Morris-Binelli et al., 2020). Williams and Jackson (2019) highlight the many different perceptual cognitive skills that contribute to team sports performance, including the ability to scan, using visual processes, in a more efficient manner in order to extract relevant cues. These authors emphasize that understanding the influence of a match on specific perceptual cognitive skills would significantly enhance team performance. Cognition, encompassing visual perception, executive function, sustained attention and working memory, influences many aspects of skill performance in team sports; however, an important component is likely due to the influence of the prefrontal cortex in monitoring errors made and shifting neural resources accordingly to positively influence responses (Arnsten, 2009). Therefore, athletes who can anticipate necessary responses successfully, overcoming the spatio-temporal constraints, are likely to be superior team sports athletes (Williams and Jackson, 2019).

Although cognitive function is often discussed as an overall phenomenon, it consists of a number of sub-components such as perception, memory, attention and executive function (Schmitt et al., 2005); all of which contribute to successful skill, and sporting, performance. A number of examples span across these domains, including the ability to recognize and recall patterns within a match, the ability to evaluate and prioritize the importance of unfolding events and using perception to scan and react efficiently to cues (Williams and Jackson, 2019). Therefore, it is important to understand the effect of team sport exercise across a range of domains of cognition. Bandelow et al. (2010) used a variety of cognitive tests, all of which had relevance to team sports players. A visual search test was used which allows for the analysis of visual perception. In a hockey specific example, perception has been highlighted (using interviews) as a key factor in goalkeeper’s abilities to successfully save penalty corner attempts (Morris-Binelli et al., 2020). Cañal-Bruland et al. (2010) assessed this using a laptop simulation of penalty corners and found visual search strategies, where gaze is shifted to the location of the ball-and-stick, enabled improved performance in the goal keepers. These findings can be applied to outfield players who are required to react to the position of the ball or opposing players in order to select the correct action. Although this research provides an insight into how cognition influences skill performance, the research assessing how the physical load, in combination with cognitive load, of sport influences cognition remains limited (Schapschröer et al., 2016). The review by Schapschröer et al. (2016) found that the impact of exercise on skill performance was influenced by the specificity of the exercise, as well as the cognitive task, with expert athletes demonstrating a positive response in speed of reaction but no change in accuracy. Hence understanding how an athlete’s cognition, across a range of domains, varies across a competitive match, will help to understand the perceptual-cognitive relationship.

The influence of exercise on executive function has also been extensively researched in laboratory-based studies (Schmit and Brisswalter, 2020). This domain is particularly important in team sports players as executive function assesses how players adaptively respond to novel stimuli and develop new strategies in order to respond accurately and effectively. Working memory, a sub domain of executive function, enables the assessment of how well a player can retrieve information from both immediate experiences, e.g., a previous interaction on the pitch, and longer term experiences, e.g., tactical information provided prior to the match (Strauss et al., 2006). Finally, sustained attention plays a role across all domains, through the ability the be able to perform skills throughout a match, despite increased mental and physical fatigue (Hajar et al., 2019). However, in order to understand how to enhance these aspects of cognition during team sport performance, it is important to understand (a) the effect of a competitive team sport match across a range of domains of cognition, and (b) the potential physiological variables responsible for these effects.

Heightened arousal, via increased secretion of catecholamines (adrenaline and noradrenaline), has consistently been named as a contributor to exercise-induced cognitive changes (McMorris and Graydon, 1997). The influence of catecholamines on cognition has traditionally followed an inverted-U relationship, whereby cognition improves up to a certain point, alongside increases in catecholamines, until over arousal began to have a negative effect on the prefrontal cortex, and resulting cognition (Arnsten, 2009). However, the inverted-U has received some criticism in recent years, with Schmit and Brisswalter (2020) suggesting the relationship is far more complex and dynamic than first assumed.

Brain-derived neurotrophic factor (BDNF) has been investigated in response to both acute and chronic exercise protocols (Winter et al., 2007; Griffin et al., 2011). Winter et al. (2007) found that increased BDNF concentration was increased following high impact anaerobic sprint intense exercise, an effect that was related to a 20% improvement in short-term learning, however, the effect in team sports is still unknown. Cathepsin B (a muscle secretory factor that is increased post-exercise) has also recently been recognized to contribute to the positive memory alterations in response to treadmill exercise (Moon et al., 2016). Contrastingly high levels of cortisol, a hormone released in response to stress (McMorris et al., 2006), have been found to detrimentally influence cognition (Kirschbaum et al., 1996). This effect is via the action of glucocorticoid receptors in the prefrontal cortex (Oei et al., 2006), resulting in the blocking of catecholamine transporters (Arnsten, 2009). Therefore, emerging evidence suggests that a number of different blood parameters (e.g., catecholamines, cathepsin B, BDNF, cortisol) can contribute to enhancing our understanding of the exercise-cognition relationship, yet the impact of team sports on cognition and blood parameters are yet to be examined together.

Cognitive function is also known to be sensitive to perceptual changes and changes in mood (Malcolm et al., 2018), two aspects which are likely to fluctuate across a team sports match. Hence, considering the range of factors that influence cognition (neurohormones, mood, and affect) is essential to a better understanding of this relationship.

Studies have tried to mimic the demands of match-play in a laboratory to investigate the influence of intermittent team sport activity on skill performance in soccer (McGregor et al., 1999) and, hockey (Sunderland and Nevill, 2005). These laboratory assessments provide us with an understanding of how high intensity intermittent exercise influences cognition; however, in order to truly understand the impact of team sport itself, an ecologically valid field-based assessment is required. To our knowledge, the only study to address this area to date assessed the influence of a field-based competitive football match on cognitive function Bandelow et al. (2010). However, this study was performed at high external temperatures (∼34°C), which presents a confounding variable due to the known effects of heat on cognition (Gaoua et al., 2011; Liu et al., 2013; Malcolm et al., 2018). Consequently, due to the challenges around collecting data in a match context, there is no study that has assessed the influence of an intermittent team sport match on cognitive function in a temperate environment, despite the importance of understanding this to facilitate the optimization of skill performance.

Therefore, the aim of the current study was to establish the influence of a competitive field hockey match on cognitive performance (perception, working memory, executive function); and to examine changes in blood parameters and mood as potential explanatory variables for these effects. It is hypothesized that response times will improve for perception and executive function tasks, with a trade-off occurring with accuracy, while the remaining cognitive tasks will be unaffected by the competitive exercise stress.

## Materials and Methods

Twenty female athletes (mean ± SD: age 19.6 ± 1.1 years, height 1.67 ± 0.03 m, body mass 64.7 ± 6.3 kg) and seventeen male athletes (age 19.8 ± 1.3 years, height 1.83 ± 0.07 m, body mass 77.9 ± 6.3 kg) volunteered for the study. The participants were recruited due to their affiliation with a hockey club and played for one of four teams across the club at an elite (7 training sessions and 2 matches per week) or sub-elite level (4 training sessions and 2 matches per week), and across all outfield positions. Ethics was gained from the institution’s invasive ethical advisory committee prior to data collection (approval code 403). All participants were provided with an information sheet and completed an informed consent form and health screen questionnaire prior to participation.

### Study Design

This study was a randomized, order-balanced, crossover design, whereby participants all completed one familiarization session (at least 1 week prior to their first main trial), one match trial and one rested control trial. Data collection across the 4 teams was between September and March. The study was order balanced by half the participants completing the control trial the day before the match, and half completing the trial the day after. Participants completed a 24 h food diary prior to the first main trial and replicated prior to the second main trial. The average temperature across the 8 trial days (control and match) was 10.3 ± 2.7°C.

#### Familiarization

Height was measured using a stadiometer (Seca 123, Seca Ltd.) to the nearest 0.1 cm and nude body mass was measured to the nearest 0.1 kg (GFK 150 AEADAM digital scale, Vitech scientific Ltd.). The participants completed a full battery of the cognitive function tests [visual search, stroop test, corsi blocks and rapid visual information processing (RVIP)] to familiarize themselves with the tests, as completed in previous research (Malcolm et al., 2018).

#### Protocol

**FIGURE 1:**
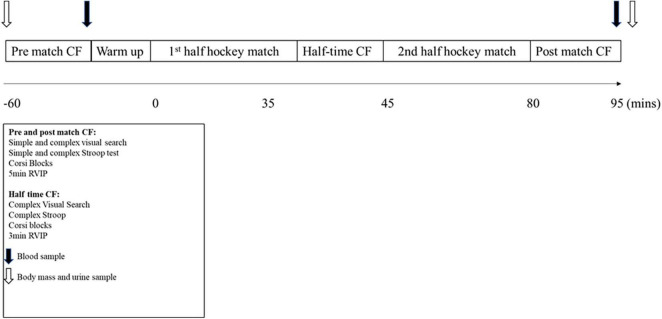
Schematic of the match day protocol. CF, Cognitive function; RVIP, rapid visual information processing. Data is mean ± SD.

Data were collected during four competitive field hockey matches and simulated control days. The participants arrived 2 h prior to the start of the match or control trial, and 2 h post-prandial. A urine sample was taken on arrival to measure urine osmolality (Osmocheck, Pocket PAL-OSMO, Japan). The participants who were deemed hypohydrated (urine osmolality > 800 mosmol.kg^–1^) were instructed to drink 0.5 L of water and re-tested in 30 min, until urine osmolality was < 800 mosmol.kg^–1^. This was completed due to the known impact of hypohydration on cognition (Judelson et al., 2007). Nude body mass was then collected in privacy, to the nearest 0.1 kg (GFK 150 AEADAM digital scale, Vitech scientific Ltd.). This was repeated following the completion of each trial (Figure 1). The change in body mass was then corrected for urine output and fluid intake to determine sweat loss and estimate hydration status.

On match day trials, the participants were fitted with a global positioning system unit (GPS) (SPI Pro, GPSports, Fyshwick, Australia) and heart rate monitor (Polar Electro, Kemple, Finland), whereas only heart rate monitors were worn on the control trials. Cognitive data was collected at three time points, relative to the timings of the match (1 h pre-match, at half time (shortened testing battery) and immediately post-match), where half time occurred after 35 min and full time following 70 min of match play. Cognitive tests were conducted in a private room immediately next to the hockey pitch. Lights were turned off, windows covered and noise canceling headphones worn to ensure no distracting stimuli occurred. All participants left the pitch immediately following the half-time and final whistle, beginning the tests within 2 min. All matches were league matches and took place in Loughborough, England between September 2015 and March 2016. The temperature and humidity were not different between control and match trials for all participants (*P* > 0.05). All timings and measurements were the same on the control trial, with the exception of the warm-up and match, which were replaced by seated rest. The participants were allowed to converse and read during the control trial rest periods, but no cognitively challenging or demanding activities were permitted.

The participants were instructed to eat as normal until 2 h prior to arrival for main trials and then not permitted to eat until the trial was completed. Water was consumed *ad libitum* during both trials (and measured), and the participants were encouraged to drink frequently during the 24 h leading to the trial to ensure euhydration. The participants were asked to avoid caffeine on the day of each trial, and avoid alcohol and exercise in the 24 h prior to each trial. The participants completed a 24 h food diary prior to their first main trial and were asked to repeat this prior to their subsequent trial in order to minimize metabolic fluctuations.

### Measurements

#### Cognitive Function Tests

A battery of cognitive function tests (visual search test, Stroop test, Corsi blocks and RVIP) was administered using a laptop computer (Thinkpad T450, Lenovo PC HK Limited, China) 1 h prior to and immediately following the competitive field hockey match. Each participant had their own laptop for the duration of the study, allowing a full team to complete their cognitive tests at the same time. Tests were always completed in the order stated above. The full version of the cognitive battery consisted of a simple and complex visual search test, a simple and complex Stroop test, Corsi blocks test and 5 min RVIP test. A shortened version of the battery of tests was administered at half time due to the time constraints, including the complex level of the visual search and Stroop tests, the Corsi blocks test and 3 min of the RVIP test. The full battery lasted ∼ 14 min and the shortened battery ∼ 7 min. Participants sat at individual desks, spaced around the room and wore noise-canceling headphones to eradicate any distracting stimuli. Prior to each test (and test level), 3–6 practice stimuli were presented to re-familiarize participants with the task and eradicate any learning effect.

#### Perception (Visual Search Test)

Perception and visual processing were assessed using the Visual Search test, as used by Cooper et al. (2015). The test is made up of two levels, with 21 stimuli on each. On the simple level of this test participants were required to react as quickly as possible to the presence of a bold, solidly outlined, green triangle. The complex level of the test required participants to complete the same task, however, the triangle shape was made up of several dots. The background was covered in green dots, which were redrawn every 250 ms, with the aim of inducing the visual effect of a flickering background. For both levels of the test the location and intervals of appearance of the stimuli, was randomized. Participants were instructed to respond to the stimuli as quickly as possible, by pressing the space bar. In order to assess visual processing and perception, this test scrutinizes the capacity to focus on specific cues whilst ignoring distracting information. The response time of correct responses and the proportion of the correct responses achieved were both measured, for each level of the test.

#### Executive Function (Stroop Test)

In order to assess executive function and selective attention, both controlled by frontal lobe function, the Stroop test was administered (Stroop, 1935). The test is comprised of two levels. The aim of the test is to assess the ability to suppress an automated response hence each level has varying amounts of interference to regulate the level of difficulty. Each level involves a test word appearing in the center of the screen, with a target word and a distractor either side of it. The target word’s position was counterbalanced for the left and right side within each test level. The participant was instructed to select the target word’s position, using the right and left arrow key, as quickly as possible once the words appeared on the screen.

Twenty stimuli were used for the simple level of the test and 40 stimuli for the complex level. On the simple level of the Stroop test, all words were presented in white font and the participant was instructed to select the word which matched the target word in the center of the screen. The color interference complex level of the test required the participant to select the word which matches with the color the word in the center of the screen was written in, rather than the word itself (which was an incongruent color). The choices stayed on the screen until the participant made a response. Following the response, an inter-stimulus interval of 1 s took place, prior to the next selection of stimuli appearing. The main outcome measures were the response time of correct responses and the proportion of correct responses.

#### Working Memory (Corsi Blocks)

Visuo-spatial short-term working memory is measured using the Corsi Blocks test (Corsi, 1972). Squares within a 3 × 3 grid are lit up in a random order. Participants are required to click on the boxes on the screen, in the order in which they lit up. Initially, three boxes are lit up in a sequence, thereafter, after each correctly remembered sequence, one additional box is lit up. If participants reached a sequence length of 9 correctly remembered boxes, the grid increased in size to 4 × 4. Performance was determined by the mean of the 3 longest correctly remembered sequences (Cooper et al., 2016).

#### Attention (Rapid Visual Information Processing)

Sustained attention is measured using the RVIP, as implemented by Hogervorst et al. (2008). The test lasted 5 min, during which the numbers between 2 and 9 appeared on the screen at 600 ms intervals. The participants were instructed to press the space bar as quickly as possible whenever detect target sequences of three consecutive odd or even numbers (e.g., “2-8-4,” “9,5,3”) appeared on the screen. There were 8 target sequences per min. For each target sequence, the participants could only respond during or within the 1,500 ms immediately following the sequence. The outcome measures were the response time of correct responses and the proportion of correct responses made.

#### Global Positioning System Unit

For the duration of the match, players wore a GPS monitor (GPS, GPSports Ltd.), mounted between the shoulder blades. GPS transmitters assessed total distance ran. Distances covered were split into 6 different speed zones; 0–4 km.h^–1^, 4–7 km.h^–1^, 7–11 km.h^–1^, 11–15.5 km.h^–1^, 15.5–20 km.h^–1^, > 20 km.h^–1^. Time spent off the pitch (e.g., during substitutions or following sending off) was not included. Data were analyzed using GPSports team AMS v.1.2.1.11.

#### Heart Rate

Heart rate monitor belts (Polar Electro Team Sport System, Kemple, Finland) were worn throughout both main trials, to provide heart rate data every 5 s.

#### Mood Questionnaire

A shortened version of the Brunel Mood Scale (BRUMS) questionnaire (Terry et al., 1999) was completed by the participants immediately prior to the first and final battery of cognitive function tests (pre-match and full-time). The participants answered 24 items linked to 6 aspects of mood; anger, confusion, depression, fatigue, tension and vigor. Each of these items was ranked on a scale of 1–5 (where 1: “not at all,” 2: “a little,” 3: “moderately,” 4: “quite a lot,” 5: “extremely”), this generated a total out of 20 for each facet of mood, which was used for analysis.

#### Blood Analyses

Due to issues with blood sample collection, not all data sets are complete. The sample size for each marker is reflected in the results. Blood samples were taken immediately following pre-match and post-match cognitive function testing, via venepuncture. This was completed within 2 min of completing the cognitive test battery, and blood was immediately treated. For serum samples, approximately 5 ml of blood was pipetted into an anticoagulant-free tube (Sarstedt, Germany) and allowed to clot for 30 min. Following this the sample was centrifuged (accuSpin 1R centrifuge, Fisher Scientific, Germany) for 10 min at 3,000 g. For plasma samples, 5 ml of blood was pipetted into each of two lithium heparin tubes (Sarstedt, Germany). Tubes were inverted five times and immediately centrifuged (accuSpin 1R, Fisher Scientific, Germany) for 10 min at 3,000 g. The supernatant for both plasma and serum was removed using a pipette, dispensed into eppendorfs and initially frozen at –20°C and then at –80°C until analyses were performed. Due to the short half-life of catecholamines, a single plasma eppendorf was snap frozen for the analysis of adrenaline and noradrenaline. Plasma was also analyzed for Cathepsin B and estrogen (female participants). Serum was analyzed for cortisol and BDNF.

All blood analysis and intra-assay CV analysis was completed by the lead researcher. Plasma was analyzed for adrenaline (*intra-assay CV: 10.5%*) and noradrenaline (*intra-assay CV: 6.5%*) using manual enzyme immunoassay (CatCombi ELISA, IBL International GmbH, Hamburg, Germany) and for Cathepsin B (*intra-assay CV: 7.6%*) via ELISA (Cathepsin B Human ELISA Kit, Abcam, Cambridge, United Kingdom). Serum was analyzed for cortisol (*intra-assay CV: 12.4%*) using an immunoassay (Cortisol, R&D systems, Abingdon, United Kingdom) and for BDNF (*intra-assay CV: 12.1%*) using separate immunoassay procedures (Human Free BDNF, R&D systems, Abingdon, United Kingdom). A 20-fold dilution was used for both cortisol and BDNF. For female participants, baseline plasma samples on the control and match day were analyzed for estrogen (estrogen, R & D systems, Abingdon, United Kingdom) in order to identify any changes in the stage of the menstrual cycle between trials.

### Data Analysis

Physiological data and corsi blocks data were analyzed using SPSS (Version 23, SPSS Inc., Chicago, Il, United States) via two-way repeated measures Analysis of Variance (ANOVA), using a trial by time approach. Where paired comparisons were required, paired samples *t*-tests with Bonferroni corrections were conducted.

The remaining cognitive data (Stroop, visual search and RVIP) were analyzed using mixed effect models in R.^1^ Response time analyses were performed using the *nlme* package (yielding *t* statistics) and accuracy analyses were performed using the *lme4* package (yielding *z* statistics), to account for the binomial nature of accuracy data. Due to the shortened battery of tests at half-time, two analyses were run. The first analysis assessed changes from pre-match to half time, and the second analysis assessed changes from pre-match to full-time. For cognitive variables, effect sizes (ES) are reported as raw effect sizes from the mixed effect models, demonstrating the magnitude of the interaction effect. Effect sizes are reported relative to the control trial (i.e., a negative effect size for response times indicates a greater improvement in response times on the match trial, a positive effect size for accuracy indicates an improvement in accuracy on the match trial). The effect size (Cohen’s d) of mood and blood parameters were calculated using *post hoc* pairings using the following thresholds: < 0.2 = trivial effect; 0.2- < 0.5 = small effect; 0.5–0.8 = moderate effect and > 0.8 = largest effect.

Pearson’s correlation coefficients were computed for correlations between absolute change in blood parameters (adrenaline, noradrenaline, BDNF, cathepsin B and cortisol) and absolute change in cognitive task performance. Correlations were also run between changes in blood parameters and match variables (e.g., GPS data). There were also run alongside change in cognitive task performance. A positive relationship will signify both variables increasing or decreasing, vs. a negative relationship where one variable increases, while the other decreases. The larger the absolute value of the correlation coefficient (e.g., closer to 1 or –1), the stronger the relationship between the variables. The magnitude of the correlation is deemed small = 0.1, medium = 0.3 and large = 0.5 (Cohen, 1992).

For all analyses, significance was set at *P* < 0.05 and data are presented as mean ± standard deviation.

## Results

### Cognitive Function

Mean data for all cognitive tests are presented in Table 1.

**TABLE 1:** Cognitive function data across the control and match day trials.

Test	Variable	Test level	Control			Match			Trial effect	Time effect	Interaction
					
			Pre	HT	FT	Pre	HT	FT			
Visual search	Response time (ms)	Simple	284 ± 24		302 ± 41	293 ± 25^∧^		298 ± 32^∧&+^	*P* < 0.01	*P* < 0.01	*P* < 0.01
		Complex	1,114 ± 190	1,079 ± 195	1,079 ± 180	1,080 ± 187	1,074 ± 202	1,051 ± 141	*P* = 0.34/0.32	*P* = 0.29/0.13	*P* = 0.99/0.92
	Accuracy (%)	Simple	98.6 ± 2.5		97.5 ± 3.7	97.7 ± 3.6		97.3 ± 3.7	*P* = 0.17	*P* = 0.09	*P* = 0.35
		Complex	97.1 ± 3.3	98.1 ± 4.3	96.4 ± 6.3	97.8 ± 5.3	97.2 ± 6.7	96.7 ± 5.2	*P* = 0.39/0.11	*P* = 0.15/0.40	*P* = 0.09/0.69
Stroop test	Response time (ms)	Simple	610 ± 82		605 ± 79	608 ± 74		625 ± 100	*P* = 0.51	*P* = 0.86	*P* = 0.09
		Complex	815 ± 173	821 ± 170	810 ± 186	827 ± 168	787 ± 163^#^	807 ± 162	*P* = 0.72/0.75	*P* = 0.60/0.48	*P* < 0.01/0.66
	Accuracy (%)	Simple	98.1 ± 3.6		97.2 ± 4.9	97.5 ± 3.8		97.1 ± 4.3	*P* = 0.60	*P* = 0.31	*P* = 0.77
		Complex	96.5 ± 4.2	95.8 ± 4.9	95.3 ± 4.9	94.5 ± 5.7	94.2 ± 5.3	95.0 ± 5.7	*P* = 0.06/0.06	*P* = 0.43/0.38	*P* = 0.62/0.34
Corsi blocks	Sequence length		6.3 ± 1.1	6.2 ± 1.0*	6.5 ± 1.0	6.3 ± 1^∧^	5.9 ± 0.9*	5.9 ± 1.1^∧+^	*P* = 0.47/0.03	*P* < 0.01/0.39	*P* = 0.09/*P* < 0.01
RVIP	Response time (ms)		471 ± 126	503 ± 57	482 ± 113	484 ± 106	466 ± 105	447 ± 123	*P* = 0.31/0.41	*P* = 0.40/0.31	*P* = 0.61/0.06
	Accuracy (%)		49.6 ± 18.4	55.9 ± 17.5*	50.7 ± 19.3	52.5 ± 18.6	52.2 ± 20.1*^#^	53.1 ± 19.3	*P* = 0.07/0.06	*P* < 0.01/0.53	*P* = 0.04/0.64

Data is mean ± SD. Pre, Baseline; HT, half-time and FT, full-time. Where tests were completed at half-time and full-time, two P-values are presented; pre to half-time and pre to full-time, respectively. Trial effect (significantly worse on the match = ^∧^), time effect (pre to half-time = * and pre to full-time = ^&^) and interaction effect (half-time con vs. Half-time match = ^#^ and full time con vs. full time match = ^+^).

#### Visual Search

**FIGURE 2:**
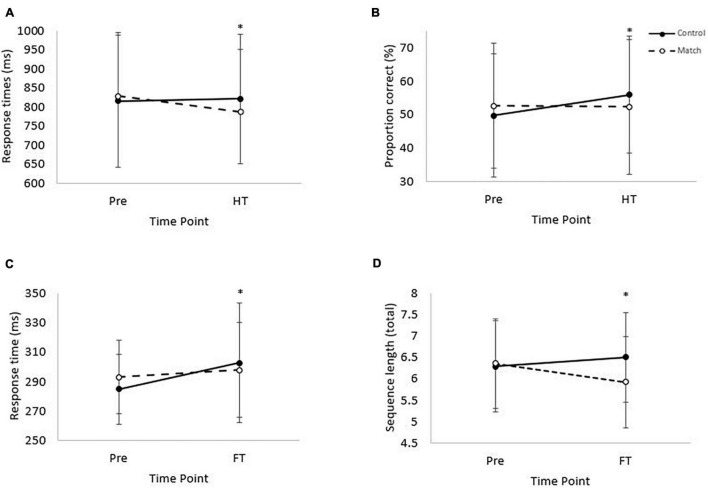
(A) Response times on the complex level of the Stroop test. Pre, prior to the match; HT, half-time. (Trial*time interaction, *P < 0.01). Data is mean ± SD. (B) Proportion correct on the RVIP test. Trial*time interaction, *P* = 0.04. Pre = baseline and HT = half-time. (Trial*time interaction, *P* = 0.04). Data is mean ± SD. (C) Response time on the simple level of the Visual Search test. (Main effect of time, *P* < 0.01; trial*time interaction, *P* < 0.01). Pre, baseline; FT, full-time. Data is mean ± SD. (D) Mean sequence length from pre-match to full-time for the Corsi Blocks test. Main effect of trial (*P* = 0.03), and trial*time interaction (*P* < 0.01). Data is mean ± SD.

There was no effect of the hockey match on response times or accuracy on the complex level of the visual search test, at half-time or at full-time (all *P* > 0.05). However, response times on the simple level of the visual search test were slower overall during the match trial [main effect of trial, *t*_(1, 3,219)_ = 2.6, *P* < 0.01; Table 1], globally slowed across time [main effect of time, *t*_(3, 3,210)_ = 5.6, *P* < 0.01] and slowed in the control condition from pre-match to full-time whereas they were better maintained in the match trial [trial*time interaction, *t*_(3, 3,210)_ = –2.9, *P* < 0.01; ES = –14 ms; Figure 2C).

#### Stroop Test

Response times and accuracy on both the simple and the complex levels of the Stroop test were not affected by the hockey match at full-time (all *P* > 0.05). However, when considering Stroop test performance at half-time, whilst response times were not different overall between the trials (main effect of trial, *P* = 0.72) or across time (main effect of time, *P* = 0.60); there was a significant trial*time interaction, whereby response times improved from pre-match to half-time on the match trial, compared to a slowing in the control trial [trial*time interaction, *t*_(3, 4,348)_ = –2.8, *P* < 0.01; ES = –44 ms; Figure 2A]. Accuracy on the complex level of the Stroop test was not affected at half-time (all *P* > 0.05).

#### Rapid Visual Information Processing

There was no effect of the hockey match on response times or accuracy on the RVIP test at full-time (all *P* > 0.05). However, at half-time, whilst response times were not affected (all *P* > 0.05), there was a tendency for accuracy to be greater on the control trial [main effect of trial, *z*_(1, 10,654)_ = 1.8, *P* = 0.07] and accuracy was greater at half-time compared to baseline [main effect of time, *z*_(3, 10,654)_ = 3.4, *P* < 0.01]. Furthermore, whilst accuracy was similar at half-time compared to baseline on the match trial, accuracy was greater at half-time compared to baseline on the control trial [trial*time interaction, *z*_(3, 10,654)_ = –2.1, *P* = 0.04; ES = –6.5%; Figure 2B].

#### Corsi Blocks

The mean length of the 3 longest remembered sequences did not differ between trials from pre match to half-time (*P* = 0.47). From pre-match to half-time sequence length decreased [main effect of time, *F*_(1, 37)_ = 7.72, *P* < 0.01], however no difference was seen between trials in the rate of change [trial*time interaction, *P* = 0.09]. The mean length of the 3 longest remembered sequences was greater in the control trial compared to the match trial [main effect of trial, *F*_(1, 37)_ = 4.83, *P* = 0.03; Table 1]. Across time performance did not change (main effect of time, *P* = 0.39). The pattern of change differed from pre-match to full-time, with recall improving on the control trial but decreasing on the match trial [trial*time interaction, *F*_(1, 37)_ = 11.89, *P* < 0.01; ES = –0.6 blocks, Figure 2D].

### Hydration Status

Urine osmolality at the beginning of the match trial (567 ± 289 mosmol.kg^–1^) and the control trial (548 ± 287 mosmol.kg^–1^) both demonstrated participants were euhydrated upon arrival, with no difference between trials (*P* = 0.73). Body mass change, corrected for fluid intake and urine output, as a percentage of resting body mass, was well maintained and similar between trials (match vs. control: 0.49 ± 1.14 vs. 0.29 ± 0.96%, *P* = 0.35).

### Characteristics of the Match

Data for distances run and heart rate across the match can be found in Table 2. There was no difference in the distances run at high speed between the first and second half for the men (*P* = 0.65) or the women (*P* = 0.81). High speed meters were positively correlated (*r* = 0.42, *P* = 0.04, *medium effect*) with change in BDNF concentration. Whereas a negative correlation was seen with change in cortisol concentration (*r* = –0.40, *P* = 0.048, *medium effect*). All other correlations (e.g., with cognitive variables) were not significant.

**TABLE 2:** Heart Rate and GPS data for male and female athletes.

	1st half	2nd half	Match
**Men**			
Mean heart rate (beats.min^–1^)	169 ± 7	164 ± 9	–
Max heart rate (beats.min^–1^)	191 ± 6	190 ± 6	–
Total distance (m)		–	6,183 ± 1,589
High speed (m)	–		502 ± 633
**Women**			
Mean heart rate (beats.min^–1^)	164 ± 19	160 ± 18	–
Max heart rate (beats.min^–1^)	195 ± 10	195 ± 9	–
Total distance (m)	–	–	5,943 ± 1,445
High speed (m)	–	–	493 ± 262

*Data is mean ± SD.*

### Mood

Raw data for all aspects of mood can be found in Table 3. Anger was greater in the match trial [*F*_(1, 35)_ = 22.90, *P* < 0.01] and increased across time [*F*_(1, 35)_ = 18.80, *P* < 0.01]. This led to a significant trial by time interaction [*F*_(1, 35)_ = 18.13, *P* < 0.01], where anger was greater post-match than following the control trial (*post hoc*, *P* < 0.01; d = 1.05, largest effect).

**TABLE 3:** Mood data for all athletes.

	Control (Pre)	Control (Post)	Match (Pre)	Match (Post)
Anger	4.4 ± 1.0	4.4 ± 1.2^&^	4.5 ± 1.2^∧^	7.3 ± 3^∧&+^
Confusion	4.6 ± 1.0	4.3 ± 0.6	4.5 ± 0.6	4.9 ± 1.8^+^
Depression	4.5 ± 1.1	4.1 ± 0.5	4.6 ± 2.5^∧^	6.4 ± 2.5^∧+^
Fatigue	8.5 ± 3.3	8.4 ± 3.3^&^	7.3 ± 2.3	11.1 ± 3.4^&+^
Tension	5.0 ± 2.4	4.4 ± 1.1^&^	5.7 ± 1.5^∧^	5.1 ± 1.6^∧&^
Vigor	9.7 ± 3.5	9.5 ± 3.9	11.3 ± 3.0^∧^	10.5 ± 3.9^∧^

*Data is mean ± SD. Trial effect (significantly greater than control = ^∧^), time effect (increased from pre to full-time = ^&^) and interaction effect (greater at full time on match vs. con = ^+^).*

A trial*interaction occurred for confusion [*F*_(1, 35)_ = 6.60, *P* = 0.02], with a higher value for confusion being demonstrated post-match than following the control trial (*post hoc P* = 0.05; *d* = 0.44, small effect).

Depression was greater in the match trial [*F*_(1, 35)_ = 17.80, *P* < 0.01] and increased across time [*F*_(1, 35)_ = 6.70, *P* = 0.01]. This resulted in a trial*time interaction [*F*_(1, 35)_ = 14.10, *P* = 0.001], with a significantly higher value for depression being demonstrated post-match than following the control trial (*post hoc P* < 0.01; *d* = 1.28, largest effect).

Fatigue increased across time [*F*_(1, 35)_ = 19.80, *P* < 0.01], causing a trial*time interaction [*F*_(1, 35)_ = 31.80, *p* < 0.01], with a higher value for fatigue following the match than following the control trial (*post hoc P* < 0.01; *d* = 0.81, largest effect). However fatigue was less at baseline [*t*_(36)_ = 2.05, *P* < 0.05] on the match day trial.

Tension was greater on the match trial (main effect of trial, *F*_(1, 35)_ = 8.00, *P* = 0.008], and decreased across time [main effect of time, *F*_(1, 35)_ = 8.00, *P* = 0.008]. However, no trial*time interaction was present, with a similar pattern of change in both trials.

Vigor was greater in the match trial than the control trial [main effect of trial, *F*_(1, 35)_ = 7.80, *P* = 0.008).

All other findings were non-significant (*P* > 0.05).

### Blood Parameters

Data for each blood parameter across the match and control trials are shown in Table 4.

**TABLE 4:** Blood parameter (mean ± SD) across the control and match day trials.

Blood parameter	Control		Match		Trial effect	Time effect	Interaction effect
		
	Pre	FT	Pre	FT			
Adrenaline (pg/ml)	96 ± 68	84 ± 48	96 ± 62^∧^	125 ± 71^∧+^	*P* = 0.04	*P* = 0.39	*P* = 0.03
BDNF (serum) (pg/ml)	23,151 ± 9,203	24,423 ± 11,183	26,617 ± 5,472^∧^	29,608 ± 5,933^∧^	*P* = 0.03	*P* = 0.15	*P* = 0.18
Cathepsin B (ng/ml)	67 ± 27	64 ± 24	64 ± 26	66 ± 28	*P* = 0.87	*P* = 0.80	*P* = 0.08
Cortisol (ng/ml)	46 ± 19	33 ± 15^&^	45 ± 17^∧^	47 ± 18^∧&+^	*P* < 0.01	*P* = 0.03	*P* < 0.01
Noradrenaline (pg/ml)	314 ± 83	348 ± 84^&^	329 ± 82^∧^	451 ± 156^∧&+^	*P* < 0.01	*P* < 0.01	*P* = 0.01

*Pre, Baseline and FT, full-time. Trial effect (significantly greater than control = ^∧^), time effect (increased from pre to full-time = ^&^) and interaction effect (greater at full time on match vs. con = ^+^).*

#### Adrenaline

Adrenaline was greater on the match trial [*F*_(1, 30)_ = 4.44, *P* = 0.04], however, no change was seen across time (*P* = 0.39). A trial*time interaction occurred [*F*_(1, 30)_ = 5.47, *P* = 0.03], where adrenaline was greater at the end of the match trial (*post hoc P* < 0.01; *d* = 0.67, medium effect).

#### Noradrenaline

Noradrenaline was greater on the match trial [*F*_(1, 28)_ = 13.04, *P* = 0.001], increased across time [*F*_(1, 28)_ = 15.05, *P* = 0.001]. There was a greater rate of increase from baseline to full-time in the match trial [trial*time interaction, *F*_(1, 28)_ = 7.72, *P* = 0.01, *post hoc P* < 0.01; *d* = 0.82, largest effect, Table 4].

#### Cortisol

Cortisol concentration was greater on the match trial [*F*_(1, 25)_ = 9.30, *P* < 0.01]. Cortisol concentration increased on the match trial and decreased on the control trial [trial*time interaction, *F*_(1, 25)_ = 22.10, *P* < 0.01]. Cortisol was greater post-match than following the control trial (*post hoc P* < 0.01; *d* = 0.85, largest effect).

#### Brain Derived Neurotrophic Factor

Overall, serum BDNF was greater on the match trial [*F*_(1, 23)_ = 5.70, *P* = 0.03, Table 4]. There was no change across time (*P* = 0.15), resulting in no trial*time interaction (*P* = 0.18).

#### Oestrogen

There was no difference between the match and control trials for estrogen concentration (control: 767 ± 426 pg.ml^–1^ vs. match: 630 ± 339 pg.ml^–1^, *P* = 0.31).

#### Cathepsin B

Cathepsin B did not differ between trials or within trial (all *P* > 0.05).

### Correlational Analysis

Correlational analysis revealed a negative correlation between the increase in cortisol and the change in Corsi Blocks performance (*r* = –0.314, *P* = 0.01, *medium*). A negative correlation occurred between the change in noradrenaline and the change in simple level Visual Search response time (*r* = –0.284, *P* = 0.01, *small*). A positive correlation was found between change in cathepsin B and the change in accuracy on the complex level of the Visual Search task (*r* = 0.22, *P* = 0.04, *small*). There were no other statistically significant correlations between the changes in blood parameters and changes in cognitive task performance (all *P* > 0.05).

## Discussion

This study was the first to isolate the effects of competitive team sports match on cognitive function. In agreement with the hypothesis, response times were improved on the match trial at half-time for the Stroop test (assessing executive function), compared to the control. However, from pre-match to half-time and full-time, working memory got worse, in comparison to the control. This study provides important implications for how cognition may be influenced across a field hockey match, suggesting a domain specific effect has occurred, and accuracy is not influenced in this process.

The findings of the present study suggest that a competitive field hockey match provides a protective influence on the simple perception test. Therefore, the increment in arousal seen with competitive sport may protect against a drop off in response time, which could be a result of a minimally arousing task being combined with rest on the control trial (Hancock, 1989). For the simple level of the executive function tasks, no changes were seen in response times or accuracy. Whereas on the complex level, response times improved at half-time on the match trial when compared to the control trial. Executive function tasks are known to be influenced by the effects of exercise, an effect likely mediated by changes in arousal (Ferris et al., 2007). From a practical standpoint, these changes suggest a player faced with opposing decisions (e.g., dribble or pass) in a game would be able to select the response more quickly. As a result of the selective permeability of the blood-brain barriers, the action of peripheral catecholamines in the central nervous system was warranted to provide a mechanistic explanation for changes seen. Noradrenaline increased across time in the match, which indicates greater activation of central nervous system (McMorris and Graydon, 1996), and likely narrowing of attentional focus (Lemmink and Visscher, 2005). Despite appearing to be domain specific, this alteration in neural function has important implications and is likely to benefit skill performance due to the constantly changing environment. In agreement with the current study, Lemmink and Visscher (2005) also showed improvements in choice reaction time following exercise, implying a narrowing of attentional focus. Despite using a cycling protocol, Lemmink and Visscher (2005) provide a physiological strain which may mimic the transient effects of a section of a match (e.g., time before a rolling substitute). The present study adds to this current literature, providing the most ecologically valid evidence regarding the effects of physiological and psychological strain associated with a team sports match on cognitive performance.

Previously, strong relationships have been demonstrated between both adrenaline and noradrenaline with choice reaction time, which highlights an inverted-U (Chmura et al., 1994). Although only small to medium effect, the correlations seen in the current study confirm that arousal facilitates improvements in perception as the change in response time was negatively correlated with noradrenaline, suggesting that noradrenaline increases alongside improvements in response time.

A decline in working memory was seen at full-time when compared to pre match. Arnsten (2009) suggested increases in noradrenaline awaken neural networks within the prefrontal cortex, influencing working memory in an inverted-U like manner. The inverted-U relationship suggests that the higher intensity, intermittent nature of the competitive sports match in the present study negated the positive effect seen following moderate exercise stress (Martins et al., 2013). A number of studies have highlighted an association between high cortisol levels and poor memory performance (Bohnen et al., 1990; Newcomer et al., 1994), where more recently Quaedflieg and Schwabe (2018) have suggested cortisol can have a positive response depending on the timing of testing in relation to the peak in cortisol. The present study found a medium negative correlation was found between changes in cortisol and working memory, reinforcing the fact that when cortisol increases, working memory decreases (Arnsten, 2009). It is believed that in states of anxiety, when cortisol concentration is increased, the brain preferentially uptakes neurotransmitters associated with emotion over those related to cognitive neurons in the hippocampus and prefrontal cortex (McMorris et al., 2006; Oei et al., 2006; Arnsten, 2009), explaining the cognitive detriment.

Serum BDNF and serum cortisol were overall greater in the match trial, compared to the control trial, which agrees with previous literature where both are greater in response to moderate intensity exercise (Vega et al., 2006). Studies have shown increases in BDNF following high impact anaerobic work to positively influence learning (Winter et al., 2007) and working memory (Griffin et al., 2011). The current findings demonstrate that throughout the match day BDNF is greater than throughout the control trial, which may result in greater BDNF, being secreted from the brain (the hippocampus) and thus enhance neuron health and cognitive performance. However, there is no significant increase across the match in comparison to the control, therefore the mechanisms associated with the change are unclear. Cortisol secretion is known to influence hippocampal neurotrophin expression and synthesis in the brain (de Assis and Gasanov, 2019) if the glucocorticoid receptors are over activated by high loads of cortisol in the blood. This suggests that the lack of change in BDNF across the match may be due to the increasing cortisol concentration throughout the match influencing it’s expression.

Mood is known to contribute to changes in cognition (Schmitt et al., 2005). Similar to Moore et al. (2012), the exercise stress in the present study resulted in increases in fatigue, anger, confusion and depression. It is understood that mood has a zone of optimal functioning (Prapavessis, 2000), hence the influence on cognition seen in the current study may indicate that effects of exercise on mood remain within this zone. Further, it has been shown, that changes in mood can enable narrowing of attentional focus, which may have also aided participants to focus on task relevant cues, responding more quickly as a result (Hüttermann and Memmert, 2015). However, the decrement in working memory (corsi blocks performance) at full time coincides with increases in anger, confusion, depression and fatigue, suggesting the domain of working memory is more susceptible to the effects of mood, likely demonstrating a more narrow zone for optimal function.

This study provides a novel approach to assessing the impact of competitive sport on cognition, however, due to the field-based nature and ecological validity, some limitations exist. Due to the time constraints at half-time, a shortened battery of the cognitive tests was conducted. It was decided to complete the more extensive battery at either side of the match despite this, due to the known differences between simple and complex tests (Gaoua et al., 2011; Moore et al., 2012) in response to stress. This process allowed us to get a more thorough understanding of the cognitive responses to competitive sport, whilst still gaining an understanding of how cognition progressed at half time. In a laboratory-based study, the variation in activity patterns between the participants could be deemed a limitation, however, due to the aim of this study being to assess the impact of competitive sport on cognition, this provides the most ecologically valid stimulus to enable us to achieve this. The study used a large sample size spread across positions in order to provide the most accurate insight possible into the physiological demands and resulting influence on cognition.

## Practical Applications

Establishing the effects of a competitive team sports match allows further investigation into both, strategies (e.g., nutritional such as caffeine supplementation) which can optimize performance, and stressors which may limit this aspect of performance (e.g., environmental). Incorporating a half-time testing session provides detail to the time course of changes in the varying domains of cognition and highlights the potential for using this break in play as an opportunity to utilize strategies for performance optimization. Although no difference was seen in match variables (high speed meters and total distance run) from the first half to the second half, future research will aim to assess additional measures (e.g., change of direction) which may place greater cognitive, neuromuscular and physiological stress upon players than running meters, and help to explain cognitive changes seen across a match.

## Conclusion

In conclusion, the present study was the first to isolate the effects of a field hockey match on cognitive function, demonstrating domain and task level (e.g., simple or complex) specific changes in cognition in response to competitive sport. These findings add to the current understanding regarding the potential explanatory variables involved in changes in team sports performance, and provides important implications as to how skill performance may be influenced in a competitive match. Future research must endeavor to elaborate on these findings by investigating the influence of a competitive sport specific protocol, as used in the current study, on sport specific cognitively challenging skills.

## Data Availability Statement

The original contributions presented in the study are included in the article/supplementary material, further inquiries can be directed to the corresponding author/s.

## Ethics Statement

The studies involving human participants were reviewed and approved by the Nottingham Trent University Invasive Ethics Committee. The patients/participants provided their written informed consent to participate in this study.

## Author Contributions

The data was collected in the field at Loughborough University, and then analyzed at Nottingham Trent University. RM, CS, and SC contributed to study design and data collection. CT and JF contributed to study design. RM, CS, SC, CT, and JF contributed to revising the manuscript. All authors approved the final manuscript and listed qualify for authorship and agreed with the order of authorship.

## Conflict of Interest

The authors declare that the research was conducted in the absence of any commercial or financial relationships that could be construed as a potential conflict of interest.

## Publisher’s Note

All claims expressed in this article are solely those of the authors and do not necessarily represent those of their affiliated organizations, or those of the publisher, the editors and the reviewers. Any product that may be evaluated in this article, or claim that may be made by its manufacturer, is not guaranteed or endorsed by the publisher.

## Footnotes

1
www.r-project.org

## References

[B1] Allard F. Burnett N. (1987). Skill in sport. *Can J Psychol.* 39 294–312.

[B2] Arnsten A. F. (2009). Stress signalling pathways that impair prefrontal cortex structure and function. *Nat. Rev. Neurosci.* 10 410–422. 10.1038/nrn2648 19455173 PMC2907136

[B3] Bandelow S. Maughan R. Shirreffs S. Ozgünen K. Kurdak S. Ersöz G. (2010). The effects of exercise, heat, cooling and rehydration strategies on cognitive function in football players. *Scand. J. Med. Sci. Sports* 20 148–160. 10.1111/j.1600-0838.2010.01220.x 21029202

[B4] Bohnen N. Houx P. Nicolson N. Jolles J. (1990). Cortisol reactivity and cognitive performance in a continuous mental task paradigm. *Biol. Psychol*. 31 107–116. 10.1016/0301-0511(90)90011-k 2103746

[B5] Cañal-Bruland R. van der Kamp J. van Kesteren J. (2010). An examination of motor and perceptual contributions to the recognition of deception from others’ actions. *Hum. Movement Sci.* 29 94–102. 10.1016/j.humov.2009.10.001 19892422

[B6] Chmura J. Nazar K. Kaciuba-Uścilko H. (1994). Choice reaction time during graded exercise in relation to blood lactate and plasma catecholamine thresholds. *Int. J. Sports Med.* 15 172–176. 10.1055/s-2007-1021042 8063464

[B7] Cohen J. (1992). A power primer. *Psychol. Bull.* 112 155–9. 10.1037//0033-2909.112.1.155 19565683

[B8] Cooper S. B. Bandelow S. Morris J. G. Nevill M. (2015). Reliability of a battery of cognitive function tests in an adolescent population. *J. Sports Sci.* 33(Suppl. 1), 41–43.

[B9] Cooper S. B. Bandelow S. Nute M. L. Dring K. J. Stannard R. L. Morris J. G. Nevill M. E. (2016). Sprint-based exercise and cognitive function in adolescents. *Prev. Med. Rep.* 4, 155–161. 10.1016/j.pmedr.2016.06.004 27413677 PMC4929070

[B10] Corsi P. M. (1972). Human memory and the medial temporal region of the brain. *Diss. Abstr. Int.* 34:819.

[B11] de Assis G. G. Gasanov E. V. (2019). BDNF and cortisol integrative system–plasticity vs. degeneration: implications of the Val66Met polymorphism. *Front. Neuroendocrinol.* 55:100784. 10.1016/j.yfrne.2019.100784 31425696

[B12] Ferris L. T. Williams J. S. Shen C. L. (2007). The effect of acute exercise on serum brain-derived neurotrophic factor levels and cognitive function. *Med. Sci. Sport Exerc.* 39 728–734. 10.1249/mss.0b013e31802f04c7 17414812

[B13] Gaoua N. Racinais S. Grantham J. El Massioui F. (2011). Alterations in cognitive performance during passive hyperthermia are task dependent. *Int. J. Hyperther.* 27 1–9. 10.3109/02656736.2010.516305 21070137 PMC3082171

[B14] Griffin É. W. Mullally S. Foley C. Warmington S. A. O’Mara S. M. (2011). Aerobic exercise improves hippocampal function and increases BDNF in the serum of young adult males. *Physiol. Behav.* 104, 934-941. 10.1016/j.physbeh.2011.06.005 21722657

[B15] Hajar M. S. Rizal H. Kuan G. (2019). Effects of physical activity on sustained attention: a systematic review. *Sci. Med.* 29:e32864.

[B16] Hancock P. A. (1989). A dynamic model of stress and sustained attention. *Hum. Factors* 31 519–537. 10.1177/001872088903100503 2625347

[B17] Hogervorst E. Bandelow S. Schmitt J. A. Jentjens R. Oliveira M. Allgrove J. E. (2008). Caffeine improves physical and cognitive performance during exhaustive exercise. *Med. Sci. Sport Exerc.* 40 1841–51. 10.1249/MSS.0b013e31817bb8b7 18799996

[B18] Hüttermann S. Memmert D. (2015). The influence of motivational and mood states on visual attention: a quantification of systematic differences and casual changes in subjects’ focus of attention. *Cogn. Emot.* 29 471–483. 10.1080/02699931.2014.920767 24865511

[B19] Judelson D. A. Maresh C. M. Anderson J. M. Armstrong L. E. Casa D. J. Kraemer W. J. (2007). Hydration and muscular performance. *Sports Med.* 37, 907–921. 17887814 10.2165/00007256-200737100-00006

[B20] Kirschbaum C. Wolf O. May M. Wippich W. Hellhammer D. (1996). Stress-and treatment-induced elevations of cortisol levels associated with impaired declarative memory in healthy adults. *Life Sci.* 58 1475–1483. 10.1016/0024-3205(96)00118-x 8622574

[B21] Lemmink K. A. Visscher C. (2005). Effect of intermittent exercise on multiple-choice reaction times of soccer players. *Percept. Mot. Skill* 100 85–95. 10.2466/pms.100.1.85-95 15773697

[B22] Liu K. Sun G. Li B. Jiang Q. Yang X. Li M. (2013). The impact of passive hyperthermia on human attention networks: an fMRI study. *Behav. Brain Res.* 243 220–230. 10.1016/j.bbr.2013.01.013 23333840

[B23] Malcolm R. A. Cooper S. Folland J. P. Tyler C. J. Sunderland C. (2018). Passive heat exposure alters perception and executive function. *Front. Physiol.* 9:585. 10.3389/fphys.2018.00585 29887804 PMC5981197

[B24] Martins A. Kavussanu M. Willoughby A. Ring C. (2013). Moderate intensity exercise facilitates working memory. *Psychol. Sport Exerc.* 14 323–328. 10.1016/j.psychsport.2012.11.010

[B25] McGregor S. Nicholas C. Lakomy H. Williams C. (1999). The influence of intermittent high-intensity shuttle running and fluid ingestion on the performance of a soccer skill. *J. Sports Sci.* 17 895–903. 10.1080/026404199365452 10585169

[B26] McMorris T. Graydon J. (1996). The effect of exercise on the decision-making performance of experienced and inexperienced soccer players. *Res. Q. Exerc. Sport* 67 109–114. 10.1080/02701367.1996.10607933 8736002

[B27] McMorris T. Graydon J. (1997). The effect of exercise on cognitive performance in soccer-specific tests. *J. Sports Sci.* 15 459–468. 10.1080/026404197367092 9386203

[B28] McMorris T. Swain J. Smith M. Corbett J. Delves S. Sale C. (2006). Heat stress, plasma concentrations of adrenaline, noradrenaline, 5-hydroxytryptamine and cortisol, mood state and cognitive performance. *Int. J. Psychophysiol.* 61 204–215. 10.1016/j.ijpsycho.2005.10.002 16309771

[B29] Moon H. Y. Becke A. Berron D. Becker B. Sah N. Benoni G. (2016). Running-induced systemic cathepsin B secretion is associated with memory function. *Cell Metab.* 24 332–340. 10.1016/j.cmet.2016.05.025 27345423 PMC6029441

[B30] Moore R. D. Romine M. W. O’connor P. J. Tomporowski P. D. (2012). The influence of exercise-induced fatigue on cognitive function. *J. Sports Sci.* 30 841–850. 10.1080/02640414.2012.675083 22494399

[B31] Morris-Binelli K. van Rens F. E. Müller S. Rosalie S. M. (2020). Psycho-perceptual-motor skills are deemed critical to save the penalty corner in international field hockey. *Psychol. Sport Exerc.* 51, 101753. 10.1016/j.psychsport.2020.101753

[B32] Newcomer J. W. Craft S. Hershey T. Askins K. Bardgett M. E. (1994). Glucocorticoid-induced impairment in declarative memory performance in adult humans. *J. Neurosci.* 14 2047–2053. 10.1523/JNEUROSCI.14-04-02047.1994 8198631 PMC6577116

[B33] Oei N. Y. Everaerd W. T. Elzinga B. M. van Well S. Bermond B. (2006). Psychosocial stress impairs working memory at high loads: an association with cortisol levels and memory retrieval. *Stress* 9 133–141. 10.1080/10253890600965773 17035163

[B34] Prapavessis H. (2000). The POMS and sports performance: a review. *J. Appl. Sport Psychol.* 12, 34–48. 10.1080/10413200008404212

[B35] Quaedflieg C. W. Schwabe L. (2018). Memory dynamics under stress. *Memory* 26 364–376. 10.1080/09658211.2017.1338299 28625108

[B36] Schapschröer M. Lemez S. Baker J. Schorer J. (2016). Physical load affects perceptual-cognitive performance of skilled athletes: a systematic review. *Sports Med. - Open* 2, 1–16. 10.1186/s40798-016-0061-0 PMC5020134 27747792

[B37] Schmit C. Brisswalter J. (2020). Executive functioning during prolonged exercise: a fatigue-based neurocognitive perspective. *Int. Rev. Sport Exercise Psychol.* 13, 21–39. 10.1080/1750984X.2018.1483527

[B38] Schmitt J. A. Benton D. Kallus K. W. (2005). General methodological considerations for the assessment of nutritional influences on human cognitive functions. *Eur. J. Nutr.* 44 459–464. 10.1007/s00394-005-0585-4 16331356

[B39] Starkes J. L. (1987). Skill in field hockey: the nature of the cognitive advantage. *J. Sports Psychol.* 9 146–160.

[B40] Strauss E. Sherman E. M. Spreen O. (2006). *A Compendium of Neuropsychological Tests: Administration, Norms, and Commentary.* Washington: American Chemical Society.

[B41] Stroop J. R. (1935). Studies of interference in serial verbal reactions. *J. Exp. Psychol.* 18 643–662.

[B42] Sunderland C. Nevill M. E. (2005). High-intensity intermittent running and field hockey skill performance in the heat. *J. Sports Sci.* 23 531–540. 10.1080/02640410410001730197 16195001

[B43] Terry P. C. Lane A. M. Lane H. J. Keohane L. (1999). Development and validation of a mood measure for adolescents. *J. Sports Sci.* 17 861–872. 10585166 10.1080/026404199365425

[B44] Vega S. R. Strüder H. K. Wahrmann B. V. Schmidt A. Bloch W. Hollmann W. (2006). Acute BDNF and cortisol response to low intensity exercise and following ramp incremental exercise to exhaustion in humans. *Brain Res.* 1121 59–65. 10.1016/j.brainres.2006.08.105 17010953

[B100] Williams A. M. Jackson R. C. (2019). Anticipation in sport: fifty years on, what have we learned and what research still needs to be undertaken? *Psychol. Sport Exer*. 42, 16–24. 10.1016/j.psychsport.2018.11.014

[B45] Winter B. Breitenstein C. Mooren F. C. Voelker K. Fobker M. Lechtermann A. (2007). High impact running improves learning. *Neurobiol. Learn. Mem.* 87, 597–609. 10.1016/j.nlm.2006.11.003 17185007

